# Regional variation in the landscape ecology of West Nile virus sentinel chicken seroconversion in Florida

**DOI:** 10.1371/journal.pone.0305510

**Published:** 2024-10-25

**Authors:** Yasmin Tavares, Jonathan Day, Bryan V. Giordano, Bradley Eastmond, Nathan Burkett-Cadena, Robert P. Guralnick, Estelle Martin, Lindsay P. Campbell

**Affiliations:** 1 Department of Ecology, Evolution, and Environmental Biology, Graduate School of Arts and Sciences, Columbia University, New York City, New York, United States of America; 2 Florida Medical Entomology Laboratory, Institute of Food and Agricultural Sciences, University of Florida, Gainesville, Florida, United States of America; 3 Department of Entomology & Nematology, Institute of Food and Agricultural Sciences, University of Florida, Gainesville, Florida, United States of America; 4 Florida Museum of Natural History, University of Florida, Gainesville, Florida, United States of America; Pasteur Institute of Iran, ISLAMIC REPUBLIC OF IRAN

## Abstract

How landscape composition and configuration impact the distribution of multi-vector and multi-host mosquito vector-borne disease systems, such as West Nile virus (WNV), remains challenging because of complex habitat and resource requirements by hosts and vectors that affect transmission opportunities. We examined correlations between landscape composition and configuration and 2018 WNV sentinel chicken seroconversion in Florida, USA across the state and within five National Oceanic Atmospheric Administration (NOAA) bioclimatic regions to understand strength and variation of landscape effects during an elevated transmission year. Although few landscape studies have examined WNV in Florida, we expected higher percentages of residential or medium-developed landscapes and more fragmented landscapes would be positively correlated with WNV seroconversion owing to the main mosquito vector habitats and avian host distributions. However, we expected to find variation in the importance of forest, wetland, and agriculture landscapes across bioclimatic regions in the state. WNV seroconversion rates were calculated using Florida 2018 Department of Health WNV sentinel chicken seroconversion data from 187 flocks maintained by mosquito control programs. Percent land cover and edge density metrics were calculated for multiple land cover classes and within multiple buffer distances from chicken coops using 2019 National Land Cover Data. We used binomial generalized linear mixed effects models to calculate the importance of landscape metrics to WNV seroconversion. We found no statewide predictors of seroconversion, but as expected, the importance of landscape varied across regions. In the north-central part of the state, we found higher seroconversion in less populated suburban areas while higher seroconversion in south-central Florida was correlated with fragmented forested areas within 0.5 km of coops and intact woody wetland areas within 2 km of coops. This work corroborates previous findings that consistent landscape predictors of WNV are difficult to identify across broader geographic areas and sets the stage for additional work that incorporates climate and landscapes interactions for a greater understanding of WNV ecology in this geographic region.

## Introduction

Landscape composition and configuration can have direct impacts on the distribution of zoonotic mosquito-borne disease systems [1]. Myriad factors, including mosquito density [2], air temperature [3], precipitation [4], humidity [5], human population distribution [6], and assemblages of hosts [7], are all elements that drive the transmission of vector-borne pathogens. However, these variables either shape the landscape (i.e., human population distribution, air temperature, precipitation) or are shaped by the landscape (i.e., mosquito density and assemblages of hosts species) [8, 9]. Thus, landscape composition and configuration serve as a proxy for geological, anthropological and climatological processes, as well as the biological communities that inhabit it. Quantifying how landscape composition and configuration impact arbovirus transmission ecology can provide a needed basis for predicting risk in a geographic area [10–13]. However, one challenge is that regional variation in the distributions of arthropod vectors and vertebrate hosts means either or both can occupy a range of differing habitats, particularly in multi-vector and multi-host systems [1]. The result is that identifying consistent and generalizable landscape drivers of transmission hazard can be challenging [14, 15], particularly across large geographic extents [16, 17].

West Nile virus (family Flaviviridae, genus Flavivirus) is one such multi-host and multi-vector zoonotic arbovirus system maintained between mosquito vectors (primarily in the genus *Culex*) and avian hosts (mainly in the Passeriformes and Columbiformes orders) [14, 18]. The virus has the broadest geographic distribution of any arbovirus, and it continues to expand in geographic range [19]. WNV is the leading cause of mosquito-borne human arbovirus disease in the U.S. and has a 10% mortality rate among infected people who develop neuroinvasive disease [20]. After its arrival to North America in 1999, substantial resources have been invested to understand the ecology of the WNV system in the U.S., including multiple studies examining landscape correlates with transmission to identify where risks may be greatest [18, 21]. However, landscape studies of WNV-positive mosquito pools or distributions of human cases have revealed differing results.

For example, in Dallas, TX urbanized areas were associated with WNV positive mosquito pools during a 2012 human outbreak where *Culex quinquefasciatus* Say (Diptera: Culicidae) mosquitoes are abundant [22]. In Chicago, IL and Detroit, MI, high incidence of WN disease in humans occurred in more suburban neighborhoods, areas with moderate vegetation, and moderate population density where *Culex pipiens* Linnaeus (Diptera: Culicidae) is often abundant [23, 24] found increased human disease risk with more fragmented habitats in Suffolk County, NY. In the southeastern U.S., urban and semi-urban landscapes have been linked to elevated WNV seroprevalence in birds in Georgia with a strong association with human housing density [21, 25, 26]. Similar results have been found in Southern California where developed landscapes and neglected swimming pools providing habitat for *Cx*. *quinquefasciatus* mosquitoes were associated with greater human incidence [27–29]. Conversely, population density had a negative correlation with WN incidence in Louisiana where *Culex nigripalpus* Theobald may play a role in transmission [30].

In other regions, agricultural land may increase transmission risk compared to adjacent areas. In California, transmission patterns were linked with fast-growing rice fields or more generic cropland preferred by *Culex tarsalis* Coquillett (Diptera: Culicidae) in the Central Valley [31]. In addition, irrigated agricultural landscapes and rural landscapes, which are the preferred habitat of *Cx*. *tarsalis*, were correlated with WNV transmission in the Pacific Northwest, Iowa, and Colorado [17, 32–34], while in South Dakota, irrigated agriculture was not correlated with human incidence [35].

In Florida, USA, little is known about landscape correlations and WNV. Florida encompasses < 1% of the WNV total reported human cases in the U.S. [36]. However, monitoring of virus activity through an ongoing sentinel chicken program conducted by the Florida Department of Health (FDOH) in conjunction with mosquito control programs indicates that the virus is endemic and transmission occurs statewide [37]. Multiple mosquito vectors and avian hosts are distributed throughout Florida. *Culex nigripalpus* and *Cx*. *quinquefasciatus* are considered the main mosquito vectors [15, 38], however additional species i.e. *Culex coronator* (Dyar & Knab) (Diptera: Culicidae) and *Culex erraticus* (Dyar & Knab) (Diptera: Culicidae) may also contribute to maintenance and/or spillover of the virus in the natural environment [39, 40]. These species occupy a range of urban and rural habitats, using artificial containers, roadside ditches, and agricultural ditches, among others as breeding sites [41, 42]. *Culex nigripalpus* and *Cx*. *quinquefasciatus* feed upon a wide variety of vertebrate hosts in Florida, including birds, mammals, and reptiles [43–45]. In addition, multiple Passeriformes birds that can serve as WNV hosts are found across a broad range of landscapes in Florida for all or a portion of the year, including American Robin (*Turdus migratorius*), Common Grackle (*Quiscalus quiscula*) [46] and Red-Winged Blackbird (*Agelaius phoeniceus*).

Despite consistent protocols for field sampling, standardized testing, and the wide distribution of the FDOH sentinel chicken program across the state, few studies have leveraged this resource to better understand the landscape ecology of WNV in Florida. Previous landscape studies focused primarily on land cover composition to predict the potential distribution of different components of the WNV system using ecological niche models [47, 48], but did not consider land cover configuration. In addition, although [49] considered edge density of multiple land cover types when analyzing 2018 WNV sentinel chicken seroconversion in northeastern Florida, no land cover variables were informative, demonstrating the need for additional analyses across multiple regions in the state.

Here, we leverage 2018 Florida Sentinel Chicken Program (FSCP) data from individual coop sites to investigate the landscape ecology of WNV sentinel chicken seroconversion in Florida. We examine landscape composition and configuration across multiple land cover types at a statewide scale and within individual bioclimatic regions in Florida. These land cover categories represent a variety of habitats for *Cx*. *nigripalpus* and *Cx*. *quinquefasciatus* mosquito densities and avian host assemblages, human population density (i.e. low, medium, and high intensity developed land cover [50], and microclimatic conditions [51, 52] (i.e. cooler shaded areas with higher relative humidity under forested canopies vs. warmer temperatures and lower relative humidity in adjacent agricultural plots receiving direct sunlight). Examination of edge habitats are representative of additional opportunities for host/vector interactions [53–55] and potential distributions of aquatic habitats important to mosquito development following precipitation events (i.e. roadside ditches) [56].

We focus on seroconversion rates for 2018, which had the greatest number of positive chickens in 10 years and were distributed broadly across the state [57]. Although little is known about specific landscapes associated with WNV seroconversion in Florida, we expect that not only the composition but the configuration of landscapes surrounding sentinel chicken coops will be important predictors of positive WNV seroconversion, including edge habitats that may affect the distribution of mosquitoes and opportunities for host interactions [54].

Overall, we expect that greater percentages of more fragmented suburban or semi-rural landscapes that support both *Cx*. *quinquefasciatus* and *Cx*. *nigripalpus* mosquito vectors and multiple WNV avian hosts will be associated with higher sentinel chicken seroconversion. However, we also expect to find regional differences in the importance of specific land cover classes to WNV seroconversion, e.g. greater agricultural land composition in central Florida will have a positive correlation with WNV seroconversion. Because the scale at which landscapes affect WNV seroconversion is unknown, we examine landscape composition and configuration summarized across multiple distances from sentinel chicken coops. Understanding these associations has the potential to reveal needed information about WNV transmission ecology in this region, while contributing to a broader understanding of multi-vector/multi-host vector-borne disease distributions across heterogeneous landscapes.

## Materials and methods

**Fig 1:**
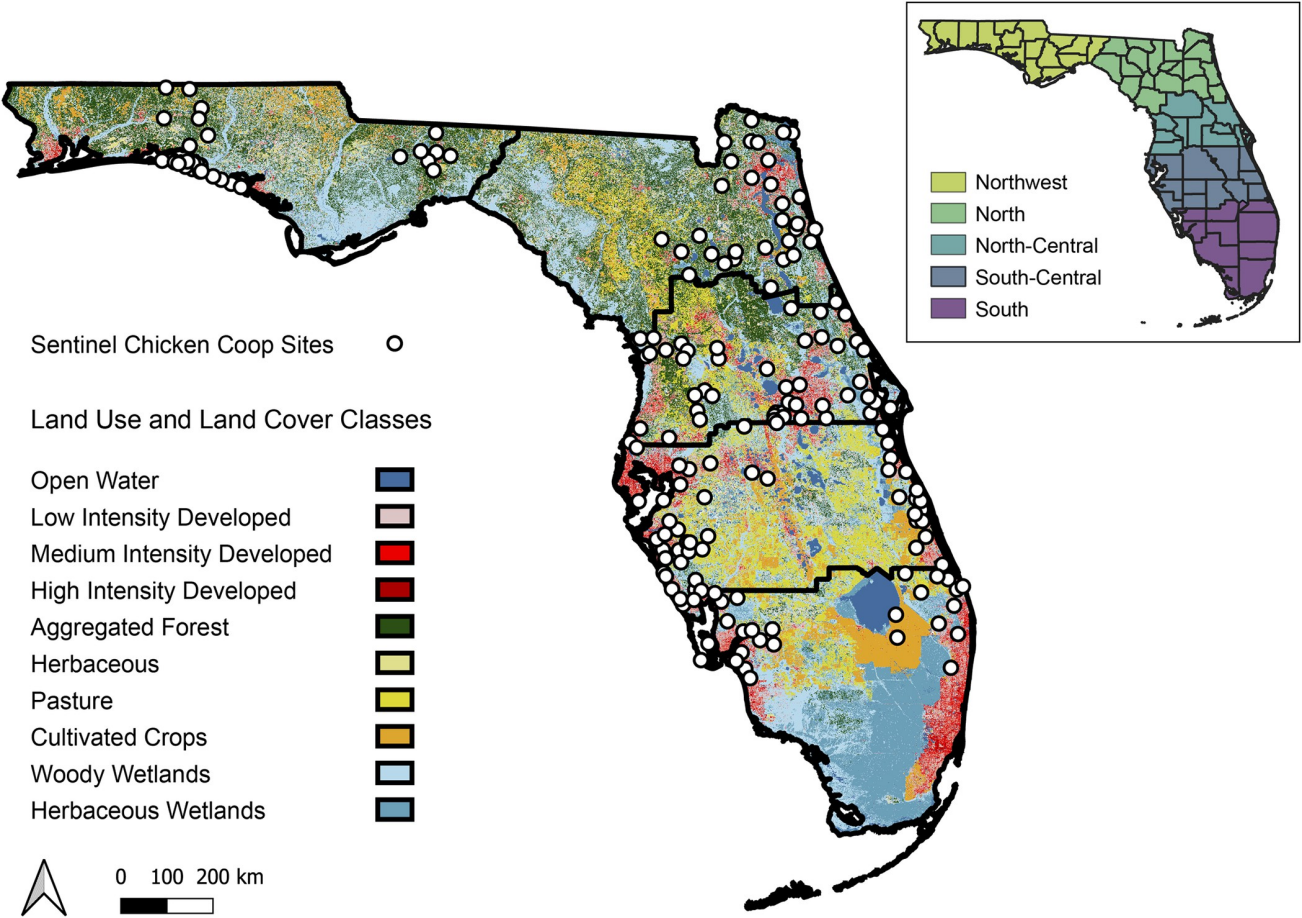
Florida map representing all active sentinel chicken coops distribution for 2018 divided into five regions (northwest, north, north-central, south-central, and south) and with the 2019 National Land Cover Database data [50]; TIGER/Line shapefile of administrative boundaries provided by the U.S. Census Bureau.

The state of Florida is predominantly peninsular and located in the southeastern portion of the United States. Florida climate is characterized as humid subtropical in the northern and central regions and as equatorial monsoonal with dry winters in the southern region of the state. In 2018, annual maximum temperatures ranged from 40°C to 42°C and annual minimum temperatures -7°C to -5°C, with 1,540 mm statewide annual average precipitation in 2018 [58]. Florida landscapes are diverse (Fig 1) including woody wetlands, forested areas, and pastures predominantly found in the northwest and north regions, while a mix of herbaceous wetlands, herbaceous agricultural and low to medium-intensity developed landscapes shape the north- and south-central regions. In the southern portion of the state, medium to high-intensity developed land cover is found along coastal areas, and cultivated crops, woody, and herbaceous wetlands dominate inland areas (Fig 1).

### Data resources used

#### Sentinel chicken monitoring data

Florida Department of Health 2018 WNV sentinel chicken seroconversion data included 187 georeferenced coop locations surveyed over a 36-week time period between April 16th and December 23rd. Blood samples from chickens are screened for flavivirus antibodies using a Hemagglutination Inhibition test, and antibody-positive birds are then tested again using an IgM enzyme-linked immunosorbent assay (ELISA) to determine whether the chickens have antibodies for WNV [37]. Weekly values at each coop were assigned a value of 0 if no chickens tested WNV seropositive and a value of 1 if at least 1 chicken tested WNV seropositive. Weekly presence/absence data was then converted to the proportion of positive weeks over the 36-week sampling period for a single value at each coop, which served as the response variable in subsequent analyses. Coop locations were then designated into five climatic regions delineated by the U.S. Climate Divisions outlined by the National Oceanic and Atmospheric Administration (NOAA) (Fig 1) [59].

*Land cover data*. United States Geological Survey 2019 National Land Cover Data was downloaded from The Multi-Resolution Land Characteristics (MRLC) consortium [50], which is available at a 30m spatial resolution. Buffers surrounding coop locations were generated at 0.5 km, 2 km, 3.5 km, 5 km, and 6.5 km, and the percentage of each land cover class and the edge density of each land cover class was calculated within the buffers. Percent land cover represented the overall composition of the land cover and edge density represented the amount of edge habitat and general fragmentation surrounding each coop site.

### Data processing and analysis

#### Variable reduction

Landscape metrics at coop sites within each region were then binned into four general classes: forest, developed, wetland, and cropland. For example, the general wetland class included landscape metrics at all buffer distances for herbaceous wetland, woody wetland, and open water. A table showing the binned classes is available in S1 Table. A conditional random forest was then run with all landscape metrics for each class and distance within the general class for the purpose of variable reduction. Conditional random forests were run using the ‘party’ package in R with ‘mtry’ values equal to the square root of the number of variables in the model and iterations ranging from 3,000 to 30,000 based on the stability of model results [50–52, 60–62]. Variable importance values were generated for each general land cover category within each region, and the variable with the greatest importance in the random forest output was selected as the environmental covariate in subsequent model runs. The result was a total of four landscape variables for each region and the state representing general forest, developed, wetland, and cropland land cover categories to be included as predictor variables in model runs. We then calculated variance inflation factor (VIF) values for environmental variables for each landscape candidate model as a test for multicollinearity.

#### Model runs and spatial autocorrelation

Binomial generalized linear mixed effects models (GLMMs) with the proportion of seropositive coops, weighted by the number of sampling weeks, served as the response variable in the regional landscape models, and the forest, developed, wetland, and cropland variables identified in the conditional random forest model served as the predictor variables in each regional model. Models included a site-level random effect to reduce overdispersion and were run using the ‘glmmTMB’ package in R and the ‘dredge’ function in the ‘MuMIn’ package was used to generate models for all combinations of variables in each candidate set [63, 64]. Models were ranked from lowest to highest Akaike’s Information Criterion (AIC) scores, AIC weights were calculated, and we used a delta AIC value of > 2 as a threshold to identify the “best” set of models [65, 66]. Residual spatial autocorrelation was investigated using the ‘Moran.I’ function in the package ‘ape’ [67]. If significant spatial autocorrelation was present, a spatial random effect term with an exponential distance decay function available in ‘glmmTMB’ was added and the candidate set was rerun [64]. Additional model diagnostics checking for non-parametric dispersion were performed in the R package ‘DHARMa’ [68]. Effect plots for variables in the top-ranking model for each region were generated using the ‘ggpredict’ function in the ‘ggeffects’ package in R [69].

## Results

Results from conditional random forests indicated variation in the importance of landscape variables across regions. Variables included in the four general land cover classes for regional candidate sets and the overall statewide model included both percent land cover and edge density variables, but varied in buffer distances and specific land cover class (S1 Table).

**Fig 2:**
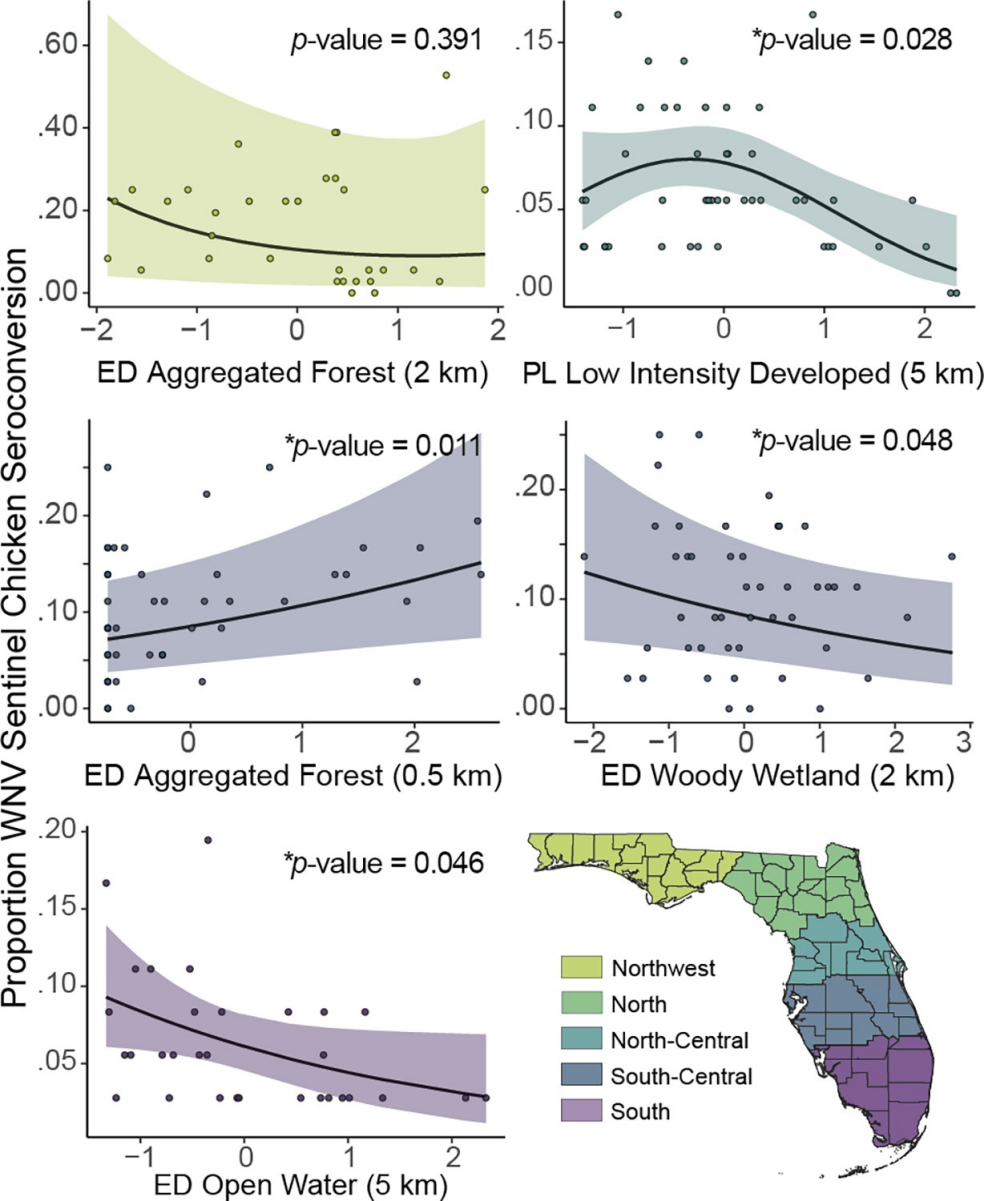
Effect plots for variables from best-performing models that demonstrate strong effects on WNV seroconversion; units are standardized values of percentages or edge densities of the land cover type. Positive values indicate greater percentages or edge density and negative values indicate lower percentages or edge density values.

As expected, results from GLMMs indicated that the importance of landscape correlations with WNV seroconversion varied across regions. However, in the statewide model and the northern most region, we did not find strong support from any of the variables included in the models, which was indicated by the intercept-only model ranking as the “best” model (Table 1). We also found weak support for correlations with edge density of forest within 2 km in the northwest region (*p-*-value = 0.391) (Fig 2). However, we found strong support for landscape composition and configuration with WNV seroconversion in the remaining regions (all other *p-*-values < = 0.05).

**Table 1:** Summary of the GLMM describing landscape associations to WNV sentinel chicken seroconversion.

Region	Variables	Estimate	Standard Error	Z Value	Pr(>|z|)	
Northwest	(Intercept)	-2.143	0.921	-2.328	0.020	*
	ED aggregated forest within 2 km	-0.278	0.191	-1.452	0.147	
	(ED aggregated forest within 2 km)^2^	0.113	0.132	0.858	0.391	
North	(Intercept)	-1.550	0.780	-1.988	0.047	*
North-Central	(Intercept)	-2.469	0.131	-18.813	<2e-16	***
	PL low intensity developed within 5 km	-0.172	0.132	-1.303	0.193	
	(PL low intensity developed within 5 km)^2^	-0.259	0.118	-2.196	0.028	*
South-Central	(Intercept)	-2.374	0.335	-7.082	1.42e-12	***
	ED aggregated forest within 0.5 km	0.251	0.099	2.544	0.011	*
	ED woody wetland within 2 km	-0.199	0.101	-1.977	0.048	*
South	(Intercept)	-2.732	0.157	-17.366	<2e-16	***
	ED open water within 5 km	-0.341	0.171	-1.996	0.046	*
All Florida	(Intercept)	-2.080	0.494	-4.211	2.54e-05	***

In the north-central region, we found a non-linear and negative effect of percentage low intensity developed land cover within 5 km on WNV seroconversion, meaning that coops surrounded by greater percentages of more rural but populated areas had lower seroconversion rates (Fig 2). Three models comprised the best set of models in the north-central region, and percentage low intensity developed land cover within 5 km was included in each model. The most parsimonious model had strong support with an AIC_w_ of 0.457 (S2 Table).

In the south-central region, we found a positive effect of edge density of forest within 0.5 km of coop sites and a negative effect of edge density of woody wetlands within 2 km on WNV seroconversion, indicating that more fragmented forests at near distances and more intact woody wetlands up to 2 km were correlated with WNV transmission (Fig 2). Four models comprised the best set of models in this region, and edge density of woody wetlands within 2 km was included in all four models, while edge density of forest was included in three models. The most parsimonious model had moderate support with an AIC_w_ of 0.227 (S2 Table).

In the south region, we found that edge density of open water within 5 km of coop sites had a negative effect on WNV seroconversion, meaning that seroconversion decreased when ocean or large bodies of water were located within 5 km of coop sites. In this region, 7 models comprised the best set of models and the most parsimonious model had the lowest support of all the regions with an AIC_w_ of 0.16. Edge density of open water was included in five of the seven best models (S2 Table).

Residual diagnostics for each of the best-performing models indicated that in the northwest and north-central regions, some non-parametric dispersion was present. Moran’s I tests for residual spatial autocorrelation indicated that all regions except for the south region required the inclusion of a spatial random effect. A summary of the best set of models for each region and the full model results are available in S2 and S3 Tables.

## Discussion

The sentinel chicken program was developed with the goal of monitoring mosquito-borne pathogen transmission in the environment to inform potential risk. Our study is the first to investigate landscape composition and configuration associations with WNV seropositive sentinel chickens across Florida, and within different bioclimatic regions. A key result is that we found no consistent statewide landscape predictors for WNV seroconversion but found clear within-state regional associations in three out of five study areas. These regional associations include significant landscape composition and configuration within near distances (0.5 km) and broader distances (2 km) from sentinel chicken coops. These results suggest that in Florida, broader extent studies may dampen relevant landscape signals present at more local to regional scales when examining landscapes alone. This work further demonstrates the many challenges with understanding and predicting zoonotic arbovirus risk in complex, human dominated systems but also provides insights about the landscape ecology of WNV in Florida and some key next steps, which we cover below.

When examining individual bioclimatic regions, we found that landscape configuration was particularly informative in the south-central region of Florida, where more fragmented forested areas near chicken coops (0.5 km) and intact woody wetlands at further distances (2 km) were positively correlated with WNV seroconversion. Within this region, chicken coops were distributed primarily in or near coastal communities within developed landscapes along the Atlantic and Gulf Coasts (Fig 1). Fragmented forested areas in this study region may be due to roadways, which are often lined with ditches for water management that support *Cx*. *nigripalpus* development [56]. In addition, these patterns can arise from dense but fragmented tree coverage within residential areas where *Cx*. *quinquefasciatus* thrives [70], and several WNV amplifying hosts are widely found in fragmented areas across these landscapes. Similar to other geographic regions, Florida is undergoing rapid and transformational land cover change with intensifying development that can result in fragmented habitats [71]. Continued investigation of mosquito vector and avian host interactions across fragmented forested habitats in the south-central region has the potential to provide additional insight into WNV ecology and transmission hazard in this area.

We also found in the south-central region that more aggregated woody wetland areas within 2 km of chicken coops were an important predictor of WNV positive seroconversion (Fig 2). This result contradicted previous findings in the northeastern U.S. and in the Chicago Metropolitan Area that found a negative correlation of percentages of woody wetland and herbaceous wetland areas with WNV transmission [16, 49, 72]. However, in addition to differences in the vegetative species comprising woody wetlands in these temperate regions, these studies focused on percentage land cover but did not examine configuration. In the south-central region of our study area, more aggregated patches of woody wetland are often associated with landscapes further from the coast, which could also be an indicator of a transition from populated areas to more semi-rural to rural areas. However, when considering distributions of WNV vector species in this region [73], demonstrated that in Manatee County, a coastal county within the south-central region, land use predicts the greatest proportion of WNV vector-competent mosquitoes in urbanized areas near the coast, and [74] found no correlation between woody wetlands and *Cx*. *quinquefasciatus* mosquitoes in New Orleans, Louisiana. Empirical field studies may yield valuable insight into how particular aspects of woody wetlands, including their configuration across the landscape, support vector-host interactions and elevate WNV distributions in this area.

The importance of landscape composition also varied regionally. In the north-central region (Fig 2), we found a non-linear effect of percentage of low-intensity developed land cover within 5 km of chicken coops. Low-intensity developed land cover includes single-family housing units mixed with vegetative cover and impervious surfaces, often in transition areas to more rural landscapes [50]. These results indicate that very high percentages of rural developed landscapes and areas that include no rural developed landscapes have low seroconversion, but that a mid-level percentage of these landscapes are a significant predictor of positive seroconversion. Overall, these results supported our expectation that more semi-urban areas would be positively correlated with WNV seroconversion. However, consistent with several other landscape studies in the U.S. [16, 21], this finding was constrained to a relatively small geographic area and similar results were not found in additional study regions in Florida. Despite the relatively small geographic area, this information has the potential to be useful to monitoring and control in this region. Additional sampling, including mosquito pool testing and blood meal analyses, will provide further opportunities to understand WNV transmission ecology within semi-rural areas in this region.

Although agricultural landscapes were correlated with WNV in multiple regions in the U.S. [17, 32–34], we did not find evidence that agricultural landscapes were correlated with WNV seroconversion at the state level or in our regional analyses in Florida, even though these habitats can support high abundances of *Cx*. *nigripalpus* and *Cx*. *quinquefasciatus* mosquitoes [41, 42]. This finding, and our overall finding of no statewide predictors of WNV seroconversion demonstrates further the challenges to generalizing results from landscape studies of the WNV system across geographic areas. The varied and nuanced relationships observed between WNV seroconversion and landscape composition and configuration in this study may be a result of the differences in the ecologies of the two putative vector species, *Cx*. *nigripalpus* and *Cx*. *quinquefasciatus* in Florida. [44] demonstrated that female numbers and class-level host use of *Cx*. *nigripalpus* and *Cx*. *quinquefasciatus* changes on an urban-to-rural gradient, but in different ways. For example, while numbers of blood-engorged *Cx*. *nigripalpus* females were relatively consistent across urban, suburban and rural sites (range 140–191), numbers of blood-engorged *Cx*. *quinquefasciatus* decreased precipitously across the same sites (386 in urban, 48 in suburban, 7 in rural). For *Cx*. *nigripalpus* the fraction of blood meals from avian hosts increased from urban to rural gradient (38.2% in urban, 54.1% in suburban, 82.1% in rural) but was relatively consistent across the urban rural gradient for *Cx*. *quinquefasciatus* (range 20.7–28.6%) [44]. These differences in the distributions and host use behaviors of the two vector species, along with variation in avian host species distributions and competency highlight challenges to disentangling landscape correlations with multi-vector/multi-host systems such as WNV.

In addition to variation in host distributions and host use among mosquito vector species, seasonal variation and heterogeneity in vector densities driven by abiotic factors, including temperature, precipitation, and humidity can affect the distribution of WNV transmission [2, 5]. These factors can also affect the timing and distribution of migration for some avian host species, and changes in avian host communities due to migratory dynamics can impact interactions between susceptible hosts and mosquito vectors, particularly across fragmented landscapes [7]. Future studies incorporating seasonal factors may provide additional information toward understanding WNV transmission ecology in Florida. While our study investigated landscape correlations with WNV transmission to sentinel chickens across a robust set of surveillance sites in Florida, some limitations exist. Here, we examined patterns resulting from a single elevated seropositive year. However, additional variation may be detected across longer time periods. Finally, we examined land cover data specific to the 2018 time period, but lagged effects of anthropogenic land cover change and ongoing landscape dynamics may also provide new insight into the ecology of WNV in Florida.

Despite these limitations, results from this study provide new information about opportunities and challenges to understanding the landscape ecology of WNV across the state. Here, we can conclude that it is beneficial to investigate the landscapes using a regional approach and that both landscape composition and configuration can be informative predictors of WNV within specific regions, when focusing on sentinel chicken seroconversion as an indicator of virus activity. Leveraging longer-term interannual time series of FDOH WNV sentinel chicken surveillance data to examine dynamic climate and landscape factors together may reveal stronger predictors of seroconversion. In addition, the inclusion of targeted field studies is needed to disentangle host and vector dynamics across these environments. This combined approach will be critical to understanding and predicting WNV transmission ecology in this region.

## Supporting information

S1 Table Variables demonstrating the greatest importance from each random forest run for developed, wetland, forest, and cropland categories in each bioclimatic region in Florida; “PL” indicates percent land cover and “ED” indicates edge density metrics. Pearson’s coefficient (*r*) values for each variable with WNV seroconversion rate weighted by number of weeks sampled.
(XLSX)

S2 Table R glmmTMB results of the “best” set of models for each bioclimatic region in Florida; “pl” indicates percent land cover and “ed” indicates edge density metrics. (XLSX)

S3 Table Values for Moran I for spatial autocorrelation calculations. (XLSX)
